# Motor Intention Quantization for Patients With Disorders of Consciousness by Multimodal BCI Combining Electroencephalography and Functional Near‐Infrared Spectroscopy

**DOI:** 10.1002/cns.70679

**Published:** 2025-12-05

**Authors:** Nan Wang, Xiaoke Chai, Jiuxiang Song, Yifang He, Qiheng He, Tan Zhang, Dongsheng Liu, Jingqi Li, Tianqing Cao, Sipeng Zhu, Yitong Jia, Juanning Si, Wenbin Ma, Yi Yang, Jizong Zhao

**Affiliations:** ^1^ Department of Neurosurgery, Peking Union Medical College Hospital Chinese Academy of Medical Sciences and Peking Union Medical College Beijing China; ^2^ Department of Neurosurgery, Beijing Tiantan Hospital Capital Medical University Beijing China; ^3^ China National Clinical Research Center for Neurological Diseases Beijing China; ^4^ School of Advanced Manufacturing Nanchang University Nanchang Jiangxi China; ^5^ School of Instrumentation Science and Opto‐Electronics Engineering Beijing Information Science and Technology University Beijing China; ^6^ Department of Neurosurgery The Second Affiliated Hospital of Soochow University Suzhou China; ^7^ Clinical College of Neurology, Neurosurgery and Neurorehabilitation Tianjin Medical University Tianjin China; ^8^ Department of Neurosurgery Tianjin Huanhu Hospital Tianjin China; ^9^ Department of Neurosurgery Aviation General Hospital Beijing China; ^10^ Hangzhou Mingzhou Brain Rehabilitation Hospital Hangzhou China; ^11^ Brain Computer Interface Transitional Research Center, Beijing Tiantan Hospital Capital Medical University Beijing China

**Keywords:** brain‐computer interface, disorders of consciousness, electroencephalography, functional near‐infrared spectroscopy, monitoring and assessment, motor intention

## Abstract

**Objective:**

The current application of single‐modality electroencephalography (EEG) or functional near‐infrared spectroscopy (fNIRS) to assess consciousness levels in patients with disorders of consciousness (DoC) has garnered significant attention. However, the diagnostic accuracy of unimodal approaches remains suboptimal. Therefore, this study aims to apply the multimodal fusion technology of EEG and fNIRS to the clinical diagnosis of DoC patients.

**Methods:**

Eleven patients with DoC (six with a minimally conscious state [MCS] and five with a vegetative state [VS]) were enrolled. The motor intention‐based brain‐computer interface (MI‐BCI) paradigm was adopted. EEG and fNIRS were recorded simultaneously. The synchronous states of EEG and fNIRS were analyzed, including time‐frequency analysis, event‐related desynchronization (ERD), and changes in oxy‐hemoglobin (HbO)/de‐oxygenated (HbR)/total hemoglobin (HbT) content. A multimodal method combining EEG and fNIRS was used to classify DoC patients.

**Results:**

The machine‐learning results of the MI‐BCI model showed that the EEG‐fNIRS multimodal approach was superior to single‐modality techniques in the diagnosis of healthy controls (HC), MCS, and VS. The multimodal model achieved a mean AUC of 0.69 ± 0.10, significantly outperforming both unimodal EEG (0.43 ± 0.19; *p* < 0.01) and standalone fNIRS (0.63 ± 0.10; *p* < 0.05). The EEG_ERD index of left‐handed MI‐BCI significantly differentiated the MCS and VS groups. Meanwhile, for the classification tasks of HC, MCS, and VS, the importance ranking of the indicators was as follows: fNIRS_ACC, EEG_ACC, fNIRS_slope, fNIRS_centroid, EEG_ERD, fNIRS_integral, and fNIRS_mean.

**Conclusion:**

The integration of multimodal MI‐BCI paradigms demonstrates clinical potential in evaluating consciousness levels, while the synergistic combination of neurophysiological and hemodynamic biomarkers provides a robust framework for enhancing the precision of bedside diagnostic protocols.

**Trial Registration:**

Clinical Trial Registry: ChiCTR2400085830

## Graphical abstract

The multimodal EEG‐fNIRS integrated brain‐computer interface (BCI) paradigm quantifies motor intention (MI) in patients with disorders of consciousness (DoC), including vegetative state (VS) and minimally conscious state (MCS). Through machine learning, spectral features (delta‐to‐gamma bands) and hemodynamic features (mean, slope, integral, centroid) are fused to decode MI strength on a weak‐to‐strong scale. This framework provides a clinically viable tool for precise consciousness assessment.

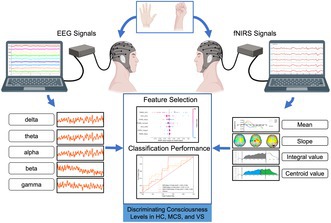

## 1 Introduction

Disorders of consciousness (DoC) can be classified as vegetative state (VS) and minimally conscious state (MCS) according to the degree of arousal, and the clinical diagnosis of consciousness grading relies on the Coma Recovery Scale‐Revised (CRS‐R), but the misdiagnosis rate reaches 40% [1, 2, 3]. Consequently, the identification of objective diagnostic biomarkers is critically important for enhancing the accuracy of DoC diagnosis [4]. A study of DoC demonstrated detectable cortical activation using task‐based functional magnetic resonance imaging (fMRI) and electroencephalography (EEG) in approximately 25% of behaviorally unresponsive patients. Among behaviorally responsive patients, one‐third exhibited cognitive‐motor dissociation (CMD), characterized by neural command responses without behavioral output [5]. Moreover, task state‐based fMRI and EEG can improve recognition rates, and both imaging techniques seem more sensitive than either technique alone. Although the excellent spatial resolution of fMRI is important for studying neural activity and cerebral blood flow changes, its large size, and high cost make it not universal [6, 7].

Functional near‐infrared spectroscopy (fNIRS) is a noninvasive test for changes in scalp blood oxygen concentration and is widely used in neurorehabilitation, stroke, neonatal, and psychiatric research [8, 9, 10]. Similar to the principle of EEG‐fMRI, the simultaneous fusion of EEG and fNIRS can capture both neurophysiological and hemodynamic activities through a neurovascular coupling (NVC) mechanism [11, 12]. EEG has higher temporal resolution and fNIRS has higher spatial resolution [6, 7]. EEG‐fNIRS multimodal technology is expected to become a new modality for DoC diagnosis due to its portability and noninvasiveness [13, 14].

The motor intention (MI) paradigm is used in subjects with normal motor function to imagine tasks in the brain as instructed but not to perform the actual movement [15]. For patients with motor dysfunction, particularly those concurrent with DoC, instructions must emphasize attempted movement execution [16]. At the earliest, Owen et al. [17] improved the misdiagnosis rate of behavioral assessment by applying MI to detect residual consciousness in patients with VS through fMRI.

**FIGURE 1:**
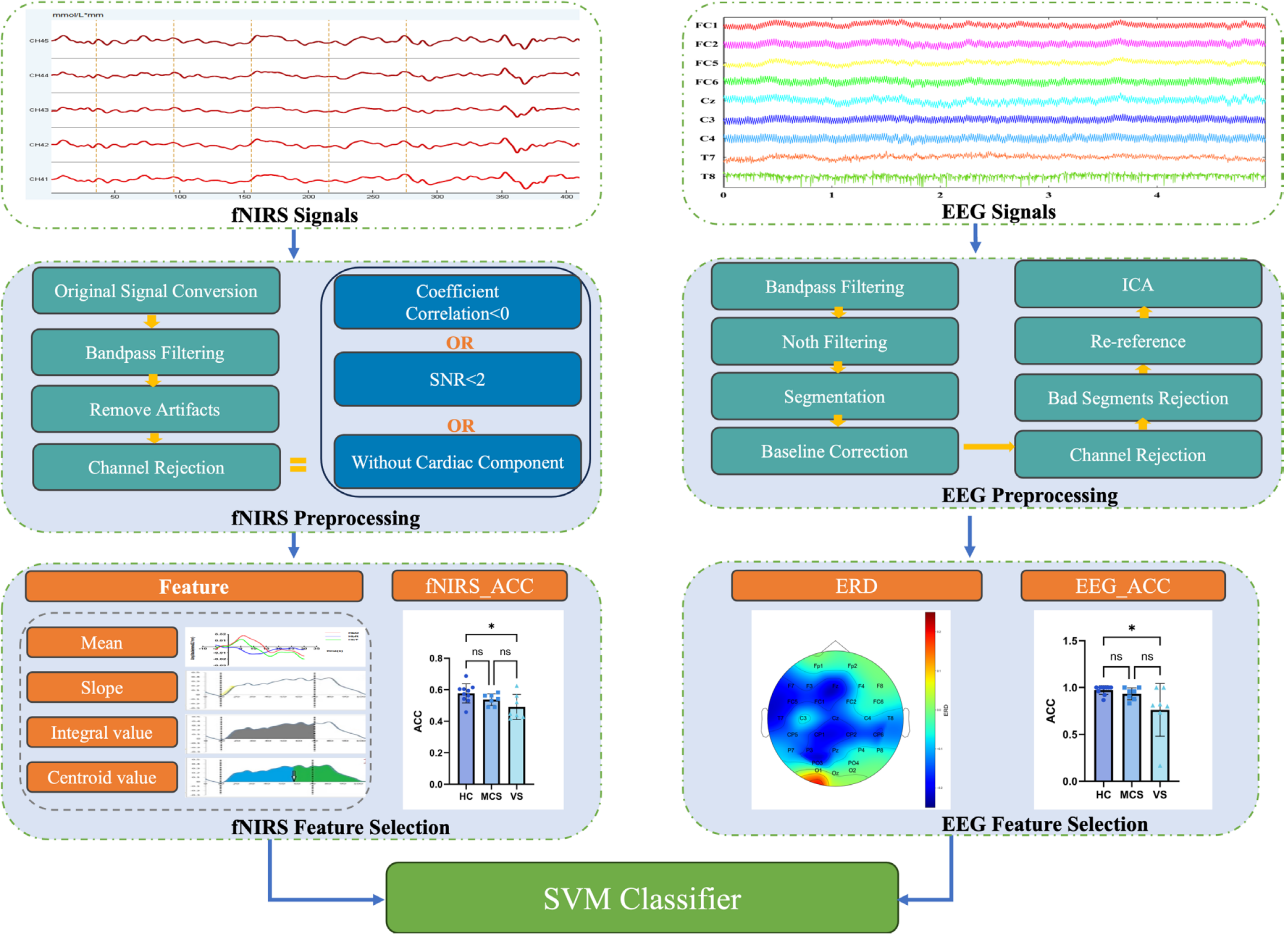
Flow chart of signal processing and classification.

In this study, we detected patients' MI through photoelectric synchronized signal acquisition combined with a brain‐computer interface (BCI) paradigm to further assess patients' state of consciousness and aid clinical diagnosis [18, 19]. This method is minimally affected by metal implants and allows for bedside recording, which largely opens up the possibility of clinical assessment [20]. The overall data analysis flowchart of this study is shown in Figure 1.

## 2 Method

### 2.1 Subjects

In this study, eight healthy subjects (six males and two females) and eleven patients (nine males and two females) were recruited from Beijing Tiantan Hospital and Hangzhou Mingzhou Brain Rehabilitation Hospital. Inclusion criteria: (1) etiology of traumatic brain injury (TBI), intracranial hemorrhage (ICH), or anoxia, etc., with a duration of more than 28 days and in a stable condition [21]. (2) diagnosis as VS and MCS according to the CRS‐R. CRS‐R scoring requires that the patient's vital signs are normal and that the patient's scoring has been performed at least 3 times within 1 week before enrollment [22]. (3) those without severe epilepsy, serious complications, and contraindications. (4) able to obtain informed consent from the legal caregivers. Exclusion criteria: (1) history of epilepsy or psychiatric or neurological disorders, (2) long‐term use of sedative or antiepileptic drugs, (3) uncontrollable infections or other serious medical diseases, (4) inability to obtain informed consent [23].

The CRS‐R, a well‐validated and widely adopted behavioral assessment tool for patients with DoC, was employed to evaluate consciousness levels [22]. The CRS‐R comprises subscales assessing auditory, visual, motor, oromotor, communication, and arousal functions. Summation of subscale scores yields a total score ranging from 0 to 23 [24]. All CRS‐R assessments were conducted exclusively by physicians with ≥ 5 years of clinical experience. Evaluations were performed only when patients were clinically stable. Consistent with established protocols, each enrolled patient underwent ≥ 3 CRS‐R assessments within the week preceding inclusion [25].

Patients were stratified into MCS and VS groups based on the CRS‐R criteria. The MCS group consisted of six male patients with a mean age of 45.5 years, and the average time since the disease began was 7.7 months. The VS group included five patients with an average age of 42.8 years, a male‐to‐female ratio of 3:2, and an average onset of 12.3 months. The clinical characteristics of the patients with DoC are shown in Table 1. Meanwhile, for the HC group, we selected eight normal individuals with a mean age of 44.75 years, M:F = 7:1, all of whom were right‐handed. In addition, written informed consent for each subject was obtained from the patient's legal guardians. The Declaration of Helsinki and all pertinent rules and regulations were followed during every procedure. The experimental protocol of this study was approved by Beijing Tiantan Hospital (KY2024‐043‐03) and passed the Chinese Clinical Trial Registry.

**TABLE 1:** Patient information.

Number	State of consciousness	Age	Gender	Duration of illness (months)	Etiology	CRS‐R
1	VS	47	M	4	TBI	000000
2	VS	21	M	7	TBI	101002
3	VS	44	F	10	BSH	000000
4	VS	37	M	10	BSH	000000
5	VS	65	F	4.5	BSH	000002
6	MCS	67	M	38.5	CI	331112
7	MCS	32	M	13.5	TBI	356323
8	MCS	64	M	8	CI	311013
9	MCS	41	M	3	TBI	230102
10	MCS	35	M	13	TBI	131102
11	MCS	34	M	1	BSH	335112

Abbreviations: BSH, brain stem hemorrhage; CI, cerebral infarction; CRS‐R, coma recovery scale‐revised; F, female; ICH, intra‐cerebral hemorrhage; M, male; MCS, minimally conscious state; TBI, traumatic brain injury; VS, vegetative state.

### 2.2 Experimental Paradigm

**FIGURE 2:**
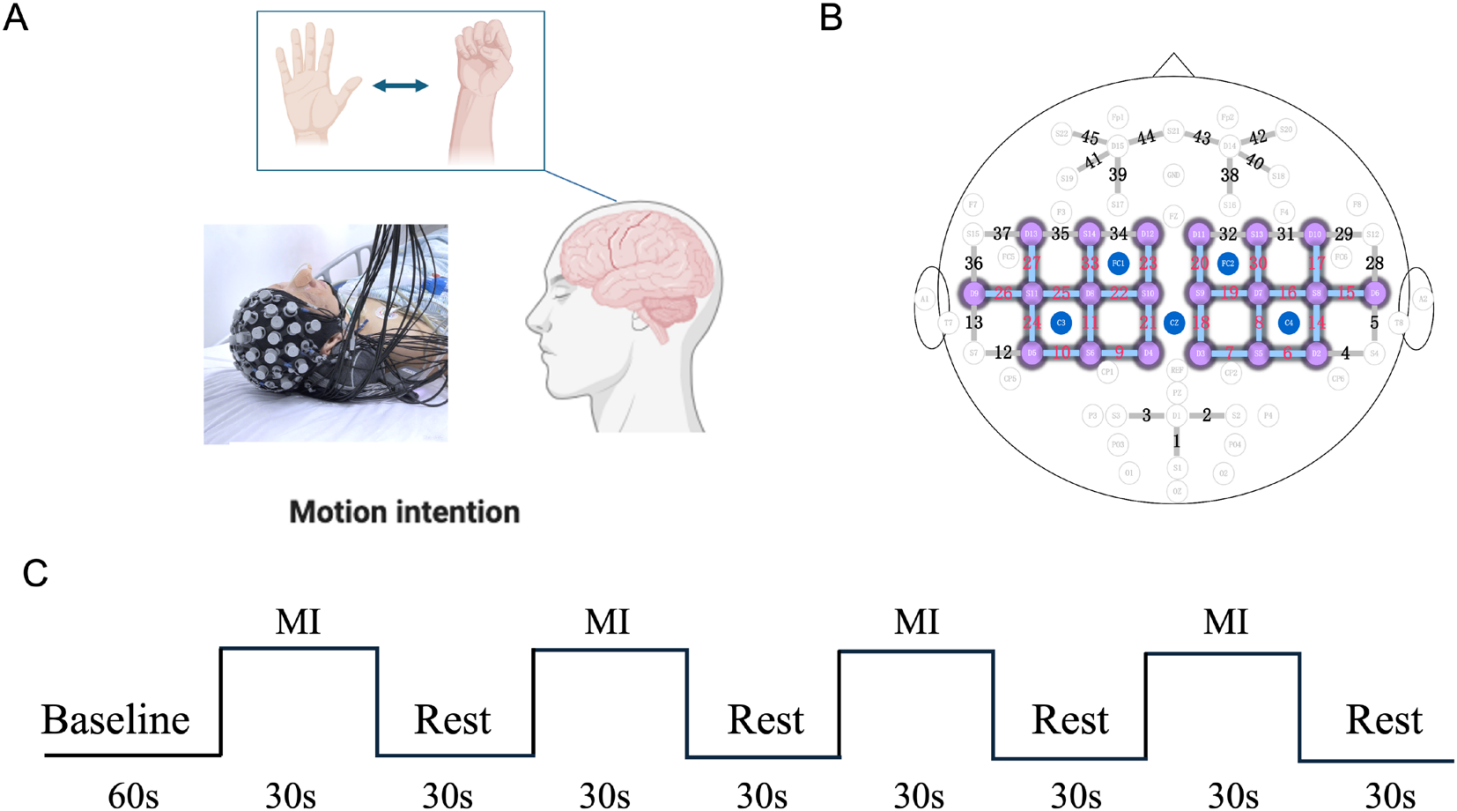
Illustration of the experimental configuration. (A) Photograph of the experimental setup. (B) Diagram of the arrangement of the optodes. (C) The experimental paradigm for the MI task.

In recent years, growing research efforts have been directed toward employing MI tasks for detecting residual consciousness in patients with DoC [13, 26, 27, 28, 29]. Figure 2 provides a schematic representation of the experimental paradigm. Our study implemented a block‐designed, command‐driven MI paradigm involving DoC patients. Participants received vocal cues through the E‐Prime 2.0 software (Psychology Software Tools, Pittsburgh, PA) instructing them to perform a command and imagine clenching their left or right fists (Figure 2A). Each experimental session comprised eight trials, systematically alternating between four left‐hand MI tasks and four right‐hand MI tasks following a standardized protocol: the experimental sequence initiated with a 60 s preparatory rest period preceding task execution (Figure 2C). This sequence was repeated four times for each limb condition, with interspersed 1‐min inter‐task intervals between contralateral MI tasks to minimize cognitive fatigue.

### 2.3 Signal Acquisition

EEG data was obtained continuously from 32 scalp channels configured with Ag/AgCl—pin electrodes. These electrodes were arranged by the standard international 10–10 system (Borecon). During the process of EEG acquisition, the subjects were maintained in an awake state. To ensure high‐quality data collection, the skin‐to‐electrode impedances were maintained below 5 KΩ, and the sampling rate was set at 1 kHz.

The fNIRS data acquisition was NirSmartII‐3000A near‐infrared brain imaging system (Jiangsu Danyang Huichuang Medical Equipment Co. Ltd.). Two wavelengths, 730 and 850 nm, were used to detect changes in oxy‐hemoglobin (HbO), de‐oxygenated (HbR), and total hemoglobin (HbT) concentrations in the brain in real time. The fNIRS system consisted of 22 sources and 15 detectors, generating a total of 45 optical channels, with a source‐detector spacing of 3.0 cm. The fNIRS optoelectronic units were arranged using a 10–10 international standard EEG system. Specifically, the 45 optical channels were symmetrically positioned in regions R_PFC (right prefrontal cortex, channels 39, 41, 44, and 45), L_PFC (left prefrontal cortex, channels 38, 40, 42, and 43), R_MC (right motor cortex, channels 9, 10, 11, 12, 13, 21, 22, 23, 24, 25, 26, 27, 33, 34, 35, 36, and 37), L_MC (left motor cortex, channels 4, 5, 6, 7, 8, 14, 15, 16, 17, 18, 19, 20, 28, 29, 30, 31, and 32), R_OC (right occipital cortex, channels 1 and 3), and L_OC (left occipital cortex, channels 1 and 2) regions in a symmetrical arrangement. The experimental structure is shown in Figure 2B. The “S” and “D” circles represent the light source and detector, respectively, and the connecting lines indicate the optical channels by numbers. The fNIRS system sampling frequency was 11 Hz.

### 2.4 Data Analysis

#### 2.4.1 EEG Preprocessing

Offline preprocessing of the EEG data was carried out using the EEGLAB toolbox within MATLAB (Version R2019a), which consisted of four distinct steps. First, abnormal EEG segments resulting from involuntary movements such as coughing and biting were identified and removed. Second, the EEG data underwent filtering to a bandwidth ranging from 1 to 45 Hz, with line noise eliminated by a 50 Hz notch filter. Third, the sampling rate of the EEG data was decreased to 500 Hz through downsampling. Fourth, the independent component analysis (ICA) function was utilized to eliminate artifacts caused by eye movements and muscle activations.

#### 2.4.2 EEG feature

Power spectral density (PSD) reflects the distribution of signal power over frequency. The PSD is calculated as follows: the signal is divided into *N* overlapping subsegments, each of which has a length of *L*, and they have *M* overlapping segments. For each sub‐segment, the power spectrum is:

(1) $$S\left(f\right)=\frac{1}{L}{\left|f\left\{x_{k}\left(t\right)\right\}\right|}^{2}$$

Then, the power spectra of all segments were averaged to obtain the final PSD estimate:

(2) $$\mathrm{PSD}\left(f\right)=\frac{1}{N}\sum_{k=1}^{N}{\mathrm{PSD}}_{k}\left(f\right)$$

Event‐related desynchronization (ERD) indicates the degree of desynchronization of signals during task execution. The reduction of brain activity in specific frequency bands is usually related to the cognitive load, attention allocation, and motor preparation of the brain during the execution of the task. The calculation of ERD is shown in Equation (3):

(3) $$\mathrm{ERD}=\frac{P_{\text{task}}-P_{\text{baseline}}}{P_{\text{baseline}}}$$

where *P*_task_ is the PSD value during the task and *P*_baseline_ is the PSD value during the rest period.

#### 2.4.3 fNIRS Preprocessing

The fNIRS data were analyzed using the NirSmart‐6000A software package. We used the NirSpark software analysis package to process the fNIRS data. Initially, the raw signals were converted into relative changes in HbO, HbR, and HbT concentrations based on the modified Beer–Lambert law. Subsequently, task‐irrelevant noise signals, including heartbeat (0.8–1.6 Hz), respiration (0.2–0.6 Hz), and blood pressure (approximately 0.1 Hz), were removed using a 0.01–0.2 Hz bandpass filter. Then, motion artifacts were identified and corrected using principal component analysis (PCA), and large, sudden motion artifact data were removed. Finally, 30s task‐state hemoglobin time series were extracted for further analysis. To ensure high‐quality fNIRS data for brain activation analysis, quality control was performed through channel pruning based on signal‐to‐noise ratio (SNR), inter‐channel correlation matrix, and spectral spectrum. Specifically, a signal channel was deemed unusable if it met the following criteria: (1) A correlation coefficient between one signal channel and another near zero; (2) A signal‐to‐noise ratio below a threshold of 2; (3) Absence of a cardiac component (~1 Hz). Channels meeting all three criteria simultaneously were excluded from further analysis.

#### 2.4.4 fNIRS feature

Four distinct hemodynamic features—the mean, integral value, centroid value and slope—were extracted from preprocessed oxygenated hemoglobin concentration changes (Δ[HbO]) for statistical analysis [30]. Feature extraction utilized two time windows defined relative to stimulus onset (*t* = 0 s):Window 1 spanned from −2 s to +30 s for calculating the mean, integral value and centroid value, while Window 2 spanned from −2 s to +5 s exclusively for slope calculation. Within Window 1, the mean was defined as the average Δ[HbO] across all sampling points; the integral value was computed as the time integral of Δ[HbO] using the trapezoidal rule; and the centroid value was derived by treating this trapezoidal area under the curve (AUC) as a mass distribution and calculating the vertical line bisecting this area (dividing it into two equal parts) based on the first moment. For Window 2, the slope feature was determined by performing a least‐squares linear regression on the Δ[HbO] data and taking the slope coefficient of the resulting regression line [31]. The integral value indicates the magnitude of the hemodynamic response during the 30s activation task, while the centroid value acts as a measure of time‐course variations throughout the task, with periods indicating the timing of the hemodynamic response [32].

#### 2.4.5 Classification of MI

Support vector machine (SVM) is a supervised learning algorithm proposed by Vapnik for solving classification problems [33]. In this study, SVM was used to classify HC, MCS, and VS. In addition, SVM was used to triple classify left‐hand MI tasks, right‐hand MI tasks, and resting states. Notably, classification accuracy for left‐hand, right‐hand, and resting state serves as a key feature for distinguishing HC, MCS, and VS. Common spatial pattern (CSP) [34] is used to extract the features of EEG signals, and the mean value, center of centroid value, slope value, and integral value are used as the features of fNIRS signals. In addition, 5‐fold cross‐validation with 10 replications was used to ensure the stability and reliability of the model, and the average result was taken as the final result. SVM models were implemented using default parameters (*C* = 1.0, kernel = “rbf”, gamma = “scale”). To maintain methodological rigor and prevent information leakage, we abstained from performing nested cross‐validation or parameter tuning within individual folds. Feature ranking was subsequently performed exclusively on the training folds using SHapley Additive exPlanations (SHAP) values. This approach ensured that feature importance assessments were derived solely from the training data distribution.

### 2.5 Statistical Analysis

Statistical analysis for this study was performed in Python (3.9.0) and SPSS. Multiple ANOVA analyses were used to verify the existence of significant differences between multiple groups. The analysis of variance test was used to verify whether there were significant differences among multiple groups. In this study, the t‐test was used to compare the differences between the MCS group and the VS group. *p* < 0.05 was considered to be statistically significant. All data in the figure are expressed as mean and standard error unless otherwise indicated.

## 3 Result

### 3.1 fNIRS Activation in the Central Region during MI‐BCI

MI related to left and right‐hand movements is primarily concentrated in the central brain regions. Based on customized photoelectric synchronous acquisition areas, we delineated the corresponding channels in the central region. Specifically, fNIRS channels were localized to the following Brodmann areas (BAs): in the postcentral gyrus, BA1 corresponds to channels 6 and 14, BA3 to channels 10 and 24, and BA43 to channels 15 and 26; in the precentral gyrus: BA4 corresponds to channels 7, 8, 9, 11, and 18, while BA6 includes channels 16, 17, 19, 20, 21, 22, 23, 25, 27, 30, and 33 (Table S1) [35].

**FIGURE 3:**
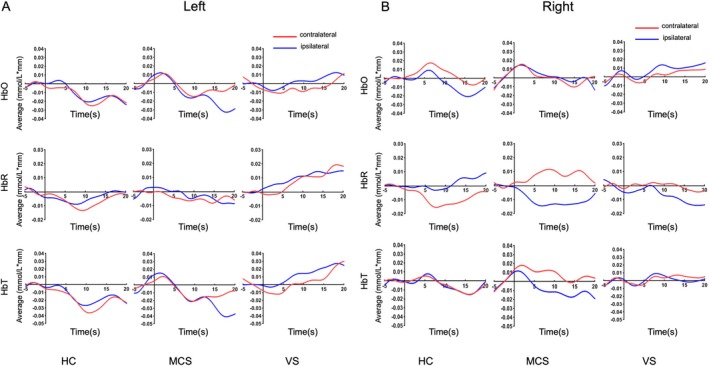
Waveform plots of HbO, HbR, and HbT in the left and right MI task for HC, MCS, and VS. For the MI condition, data from all four MI blocks were averaged by fNIRS. Means and standard error of the means for the contralateral hemisphere, concerning the spatial location of the graphic, are visualized in blue, and for the ipsilateral hemisphere in red. (A) Grand average of HbO concentration (top), HbR concentration (middle), and HbT concentration on the contralateral (red) and ipsilateral (blue) side evoked by left‐hand movement intention in HC, MCS, and VS. (B) Grand average of HbO concentration (top), HbR concentration (middle), and HbT concentration on the contralateral (red) and ipsilateral (blue) side induced by right‐hand movement intention in HC, MCS, and VS. Image data were averaged for the calibration area.

Figure 3 illustrates the mean concentration changes in HbO, HbR, and HbT from 0 to 20 s during left‐hand and right‐hand MI tasks for the HC, MCS, and VS groups, distinguishing between ipsilateral and contralateral responses. Descriptively, for HbO concentration, the HC group showed trend‐consistent concentration changes ipsilateral and contralateral to the right‐handed MI throughout the entire task period. Additionally, right‐handed MI showed stronger activation on the contralateral side than on the ipsilateral side, with contrasting ipsilateral and contralateral oxygenation changes. In the MCS and VS groups, the primary response time was between 5 and 15 s, with results indicating stronger ipsilateral oxygenation changes, reflecting the difference in consciousness levels between DoCs and HC subjects. Furthermore, the difference in oxygenation changes between ipsilateral and contralateral responses was relatively small.

**FIGURE 4:**
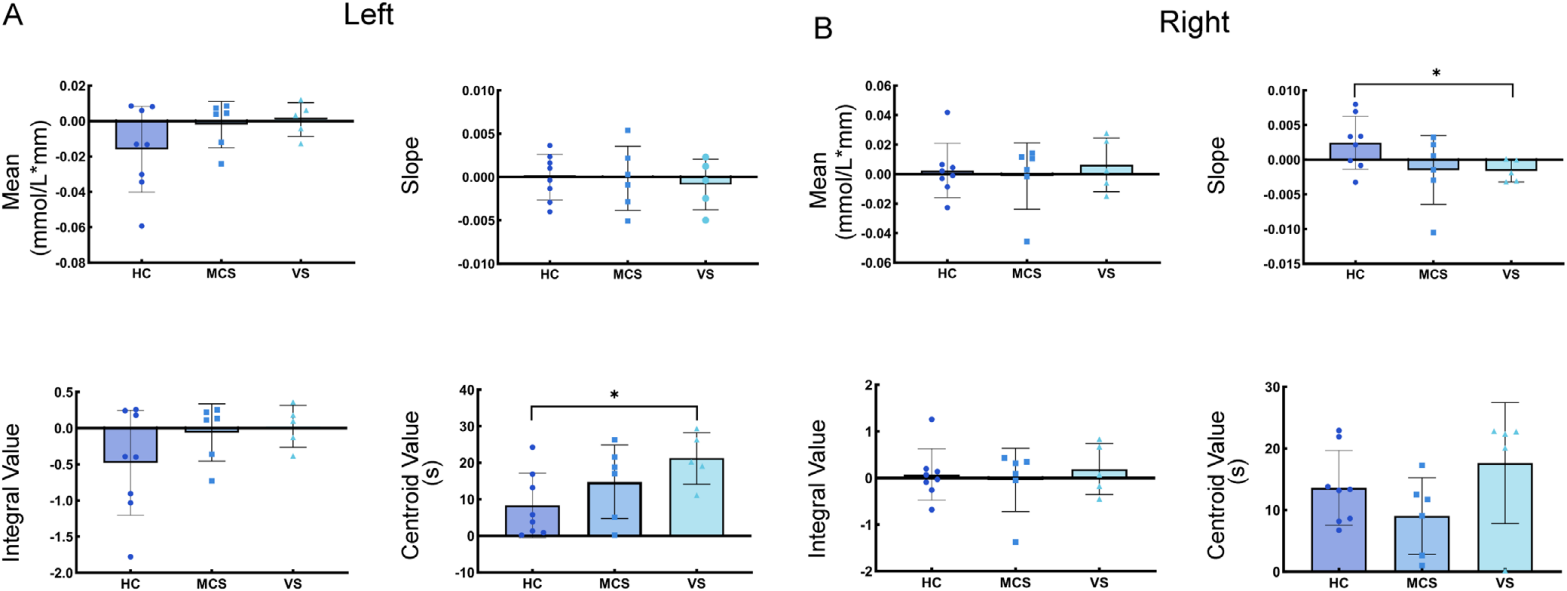
Mean, integral value, centroid value, and slope of HbO during left and right MI task for HC, MCS, and VS. Statistical plots of fNIRS activation for (A) left‐ and (B) right‐handed MI in the HC, MCS, and VS groups. *p < 0.05.

The fNIRS technique is based on measurements of hemodynamic changes in cerebral blood flow. HbO, HbT, and HbR concentrations were quantified based on their respective peak amplitudes. In this study, we compared the differences in the characteristic indicators of brain activation (mean, slope, integral value, and centroid value of HbO change) in the HC, MCS, and VS groups, and conducted statistical analysis by independent sample *T*‐test. Statistical analysis of the activation characteristics using fNIRS revealed that the center of centroid effectively distinguished between HC and VS in the left‐hand MI task (Figure 4A), while the slope value effectively differentiated HC from VS in the right‐hand MI task (*p* < 0.05) (Figure 4B). Our results show that fNIRS detection of hemodynamic features of brain activation, in particular quantitative analysis of the center of centroid values and slope features, showed significant discriminatory power in distinguishing between the level of consciousness of HC, MCS, and VS groups.

### 3.2 Spatial Distribution of fNIRS Activation during MI‐BCI

**FIGURE 5:**
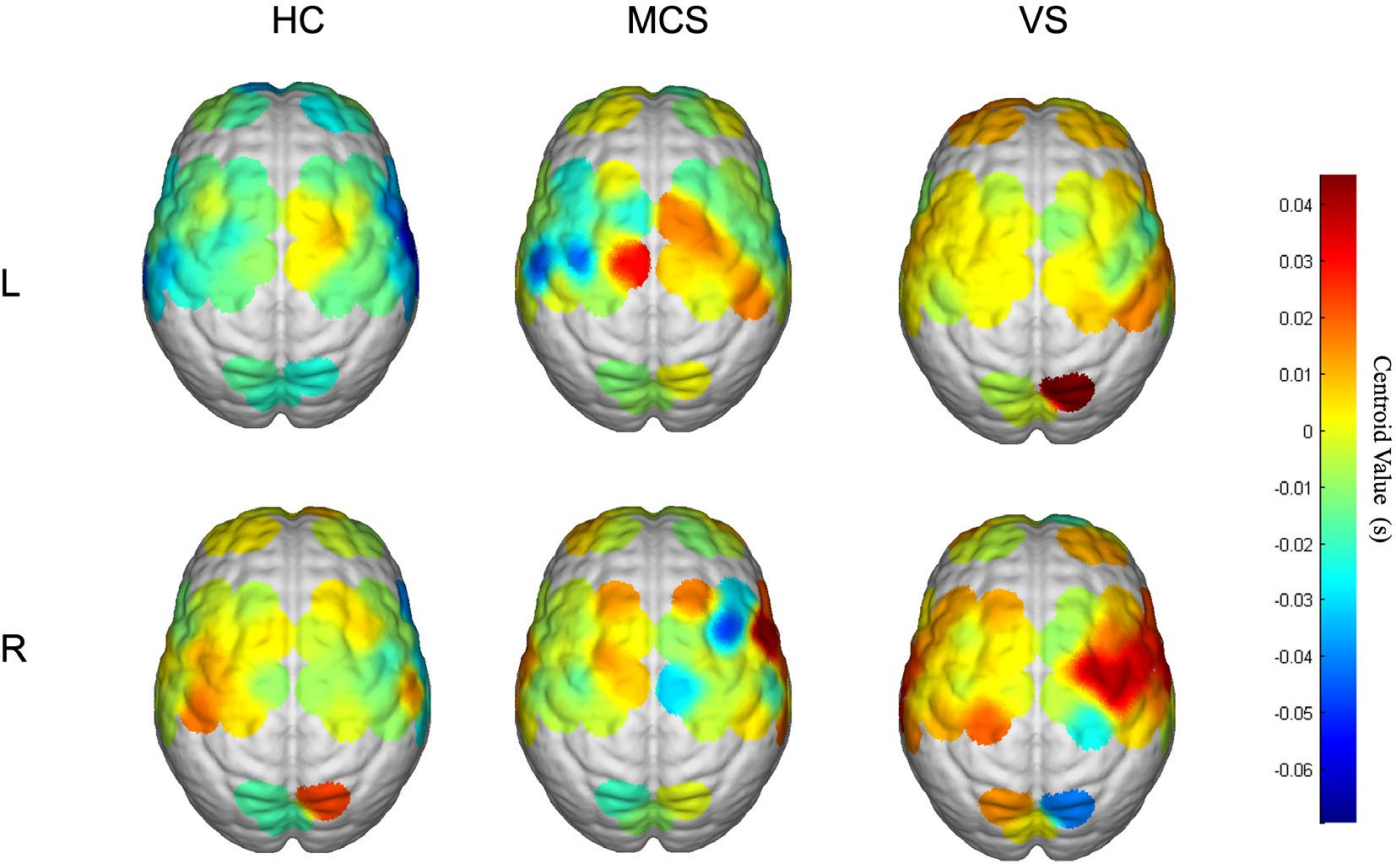
Cortical mapping of fNIRS‐HbO centroid value in HC, MCS, and VS groups. Activation levels in the left‐handed and the right‐handed MI task (*p* < 0.05, FDR). The graph shows the centroid value in the brain. The centroid value changes in HbO concentration induced by HC, MCS, and left‐hand motion intent (top). The slope change of HbO concentration caused by HC, MCS, and vs. right‐hand MI (bottom). The image data in the correction area was averaged. For the same task, the closer the color is to red, the longer the activation time, and the closer the color is to blue, the shorter the activation time.

Figure 5 illustrates the topography of the central brain region according to HbO center of centroid changes for left‐handed MI‐BCI and right‐handed MI‐BCI. In fNIRS, the center of centroid value reflects the response rate of blood oxygenation changes during brain activation. As shown in the figure, more pronounced brain activation due to left‐handed MI than right‐handed MI was observed in the movement region in HC. This suggests that more time and energy expenditure is required for motor activation with the nondominant hand. In addition, the topographies of the HC, MCS, and VS groups show that VS is greater than MCS, which is greater than the benefit, which suggests that the activation speed of the three populations is in the order of VS group < MCS group < HC group, which indicates that the neural response speed of the VS patients is slower than that of the MCS patients, which in turn is slower than that of the healthy control group. After false discovery rate (FDR) correction, these correlation results were significant.

### 3.3 EEG Time‐Frequency Spectra during MI‐BCI Task State

**FIGURE 6:**
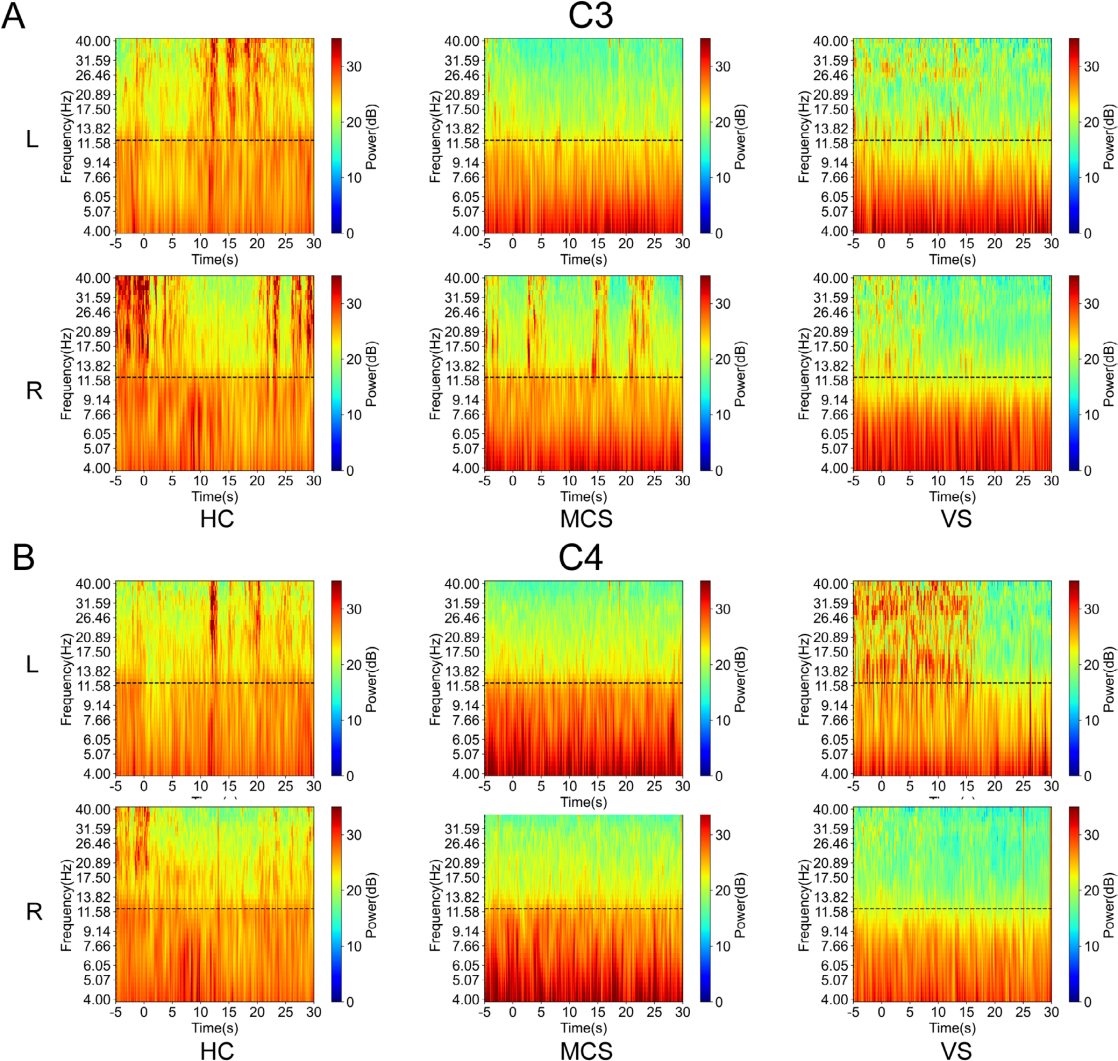
TFRs of EEG signals during left‐hand and right‐hand MI tasks across HC, MCS, and VS groups. (A) TFRs at C3 electrode (left sensorimotor cortex); (B) TFRs at C4 electrode (right sensorimotor cortex).

To analyze the differences in the complexity of different brain regions during MI, the time‐frequency representations (TFRs) of the subjects in the stimulus‐side (C3 electrode) and their contralateral (C4 electrode) brain regions during the execution of the MI task were calculated separately. Figure 6 presents the TFRs of healthy individuals, MCS, and VS patients from ‐5 to 30s during MI tasks. Comparative analyses revealed distinct oscillatory patterns among HC, MCS, and VS. Topographic specificity was observed, wherein Figure 6A illustrates spectral dynamics over the left sensorimotor cortex (C3 electrode), while Figure 6B displays corresponding responses over the right sensorimotor cortex (C4 electrode). In healthy individuals, overall energy fluctuations are more pronounced above 12 Hz, whereas in MCS patients, persistent energy activation is observed in the 8–12 Hz range, but activation above 12 Hz is less prominent. In contrast, VS patients exhibit sustained energy activation only below 8 Hz. The time‐frequency spectrum indicates that not all groups consistently exhibit MI‐related components throughout the entire 30s paradigm.

### 3.4 ERD during MI‐BCI task

**FIGURE 7:**
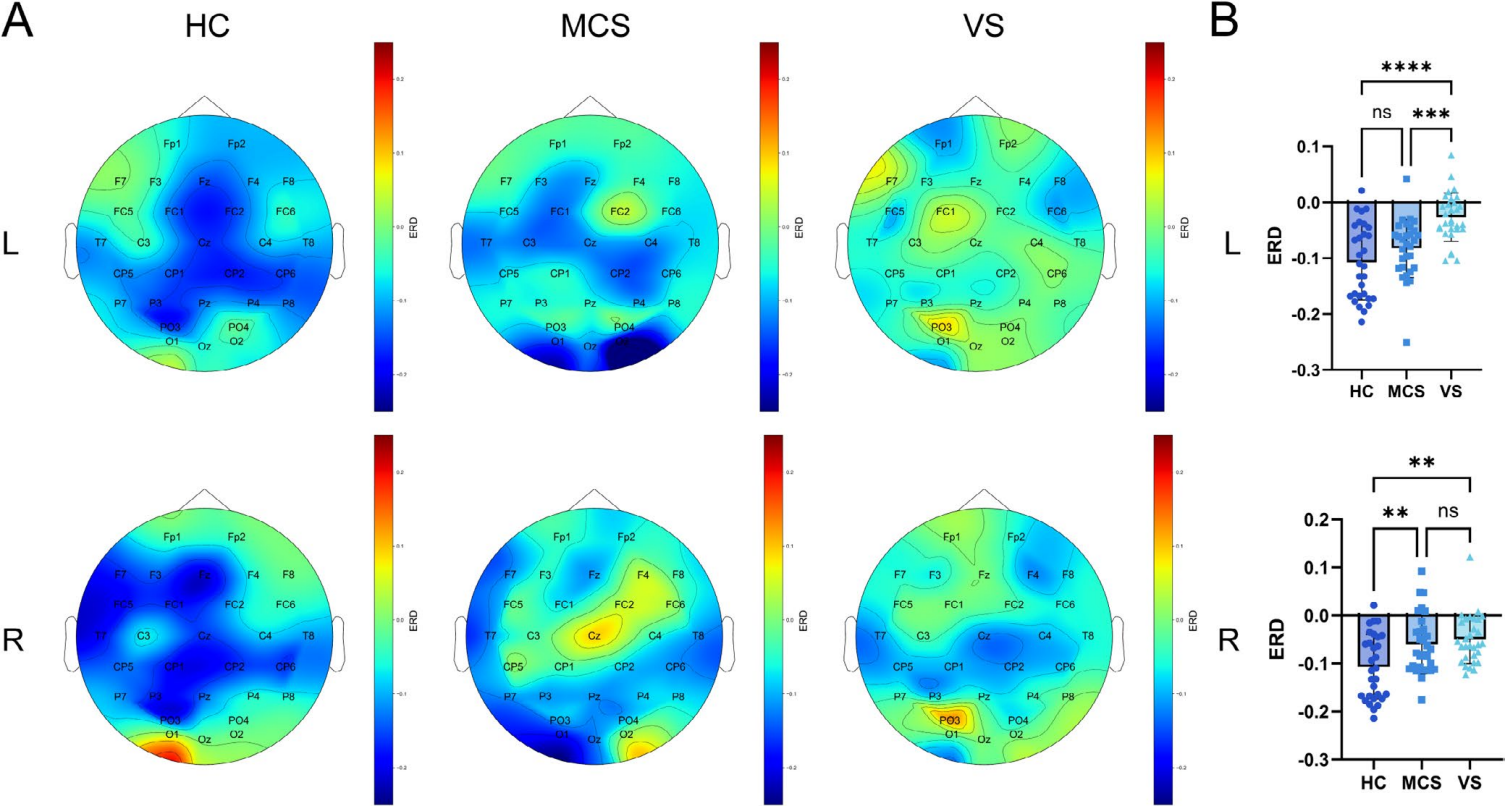
ERD topographic maps during the MI‐BCI task. (A) Left: HC group. Middle: MCS group. Right: VS group. Top: Left‐hand MI task. Bottom: Right‐hand MI task. These changes were induced by the MI of the task stimulus. (B) Significant differences in ERDs between groups in the left‐ and right‐handed MI conditions. **p < 0.01, ***p < 0.001, ****p < 0.0001.

To quantify ERD, we analyzed left‐hand and right‐hand MI tasks across HC, MCS, and VS groups. As illustrated in Figure 7A, the HC group exhibited distinct ERD patterns in the central cortical regions corresponding to their executed movements during MI tasks. In contrast, the MCS group demonstrated reduced spatial activation extent in these regions, while the VS group showed further diminished ERD magnitude and distribution. Analysis revealed that healthy controls displayed characteristic contralateral hemispheric dominance (left hemisphere activation during right‐hand MI and vice versa), whereas both MCS and VS groups exhibited abnormal lateralization patterns. Statistical comparisons confirmed significant between‐group discrimination (HC vs. VS) in both hand MI conditions. Notably, left‐hand MI ERD metrics demonstrated exceptional sensitivity in distinguishing MCS from VS patients (*p* < 0.05) (Figure 7B). These neurophysiological findings provide critical biomarkers for preclinical diagnosis of DoC.

### 3.5 BCI‐MI task ACC for Different Levels of Conscious State

**FIGURE 8:**
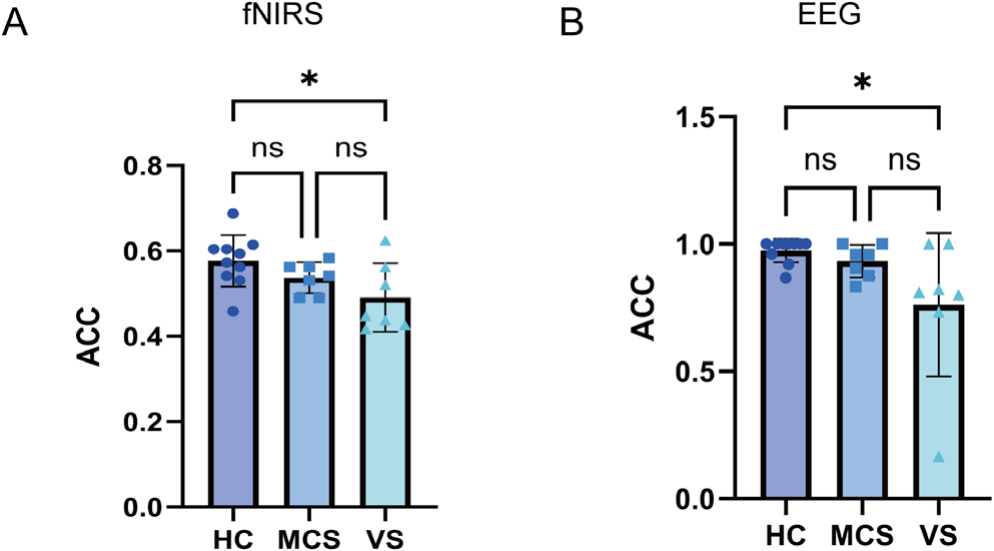
Classification accuracy of MI task recognition in HC, MCS, and VS groups. (A) ACC of MI task recognition using fNIRS across HC, MCS, and VS. A significant difference in decoding accuracy was observed between the HC and VS groups (*p < 0.05). (B) ACC of MI task recognition using EEG across HC, MCS, and VS. A significant difference in decoding accuracy was observed between the HC and VS groups (*p < 0.05).

One of the key objectives of our study was to examine whether different levels of conscious states influence the recognition of MI tasks. To this end, we classified resting‐state, left‐handed, and right‐handed MI tasks across three distinct consciousness levels (HC, MCS, and VS). As illustrated in Figure 8A, classification using fNIRS resulted in accuracies of 57.71% for HC, 53.72% for MCS, and 49.11% for VS. Statistical analysis revealed a significant difference between HC and VS (*p* < 0.05), whereas no significant differences were observed among the other groups. In contrast, classification using EEG signals yielded accuracies of 97.47% for HC, 93.28% for MCS, and 76.19% for VS (Figure 8B).

### 3.6 Classification of Different Levels of Conscious State by BCI feature

**FIGURE 9:**
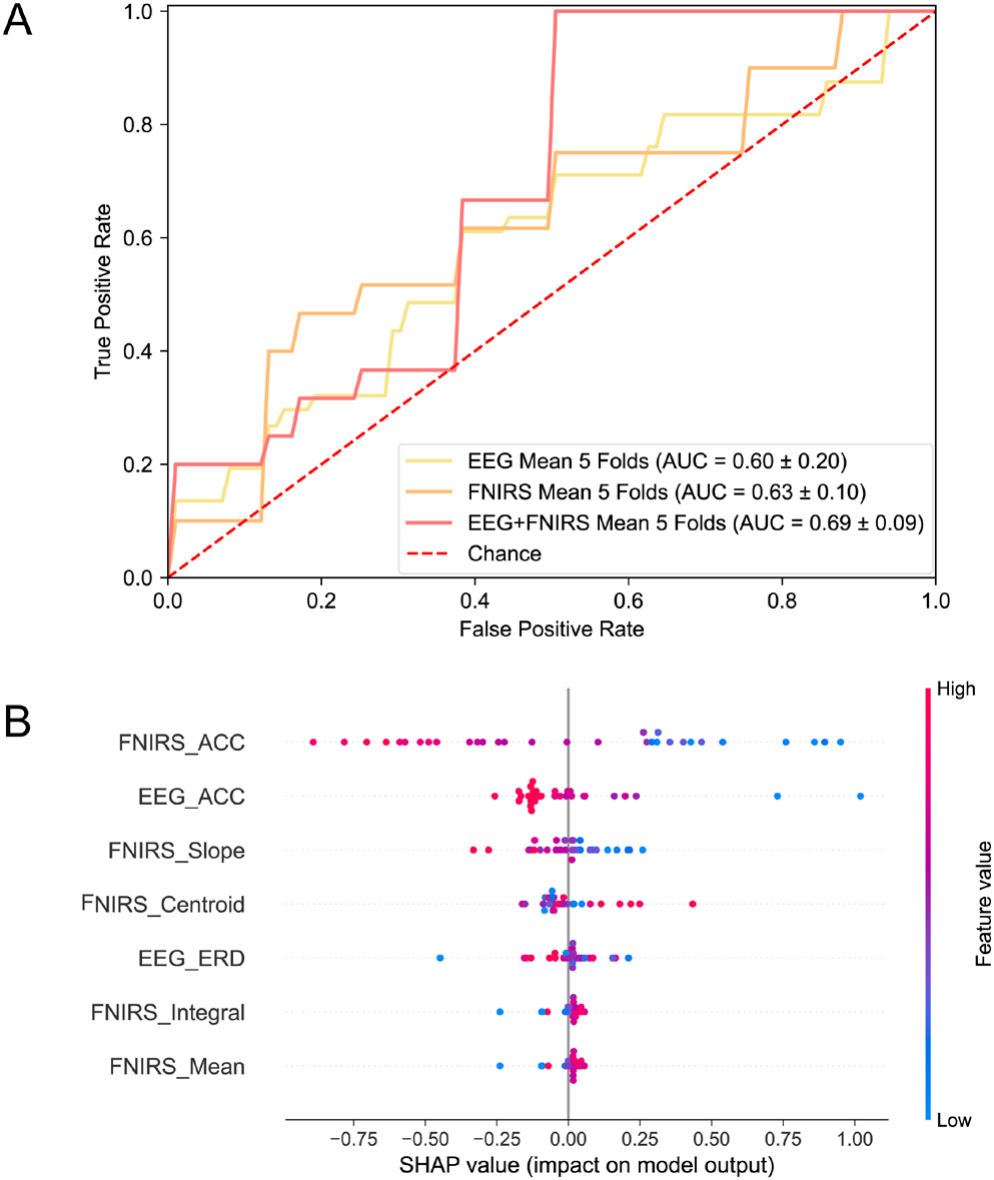
Classification of levels of impaired consciousness and feature ranking of patients. (A) The multimodal EEG‐fNIRS classifier demonstrated a superior three‐class. Feature classification of HC, MCS, and VS based on moving right‐hand MI. Yellow solid lines indicate EEG unimodal classification results, gold solid lines indicate fNIRS unimodal classification results and pink solid lines indicate EEG‐fNIRS bimodal classification results. The level of classification accuracy is indicated by the AUV value. (B) Feature ranking. The fivefold class approach determines the ordering of features for the three population classifications of HC, MCS, and VS. The feature importance is: FNIRS_ACC, EEG_ACC, fNIRS‐slope, fNIRS‐centroid, EEG_ERD fNIRS_integral, fNIRS _mean. fNIRS_ACC and EEG_ACC refer to the accuracy under the MI task.

The multimodal EEG‐fNIRS can effectively identify the level of impaired consciousness. Compared with the unimodal approach, the multimodal EEG‐fNIRS classifier shows superior three‐class discrimination performance (HC vs. MCS vs. VS). As shown in Figure 9A, the multimodal model achieved a mean AUC of 0.69 ± 0.10, significantly outperforming both unimodal EEG (0.43 ± 0.19; *p* < 0.01) and standalone fNIRS (0.63 ± 0.10; *p* < 0.05). Notably, the multimodal approach exhibited reduced outcome variability (SD = 0.10) compared to EEG‐based classification (SD = 0.19), indicating enhanced clinical reliability. The SHAP‐based feature importance analysis method (Figure 9B) identified five key discriminators for conscious state differentiation: fNIRS_ACC (classification accuracy of MI task by fNIRS), EEG_ACC (classification accuracy of MI task by EEG), fNIRS_slope, fNIRS_centroid, and EEG_ERD.

## 4 Discussion

This study employed the dual‐modality technology of EEG and fNIRS to synchronously collect data from HC, MCS, and VS subjects during left‐hand and right‐hand MI‐BCI paradigms. EEG analysis primarily focused on the C3 and C4 brain regions [36], while fNIRS analysis was centered on the motor cortex [37]. To ensure temporal synchronization, averaged epochs from 0 to 30 s post‐task onset were simultaneously extracted for EEG‐fNIRS data analysis. In the three‐class classification of resting state, left‐hand MI, and right‐hand MI, fNIRS exhibited suboptimal performance in MI task recognition, with significantly lower classification accuracy compared to EEG. From the perspective of motor paradigms, EEG demonstrates greater sensitivity than fNIRS in assessing the intensity of residual MI, making it a more reliable indicator in this context [38]. The three‐class classification results of HC, MCS, and VS showed that the multimodal model achieved a mean AUC of 0.69 ± 0.10, significantly outperforming both unimodal EEG (0.43 ± 0.19; *p* < 0.01) and standalone fNIRS (0.63 ± 0.10; *p* < 0.05). The EEG‐fNIRS multimodal approach enhances the classification accuracy of consciousness levels. Likely due to the complementary nature of EEG electrical signals and fNIRS metabolic signals in both temporal and spatial domains [39]. Regarding the reliability of MI‐BCI classification metrics for different levels of consciousness, the present study demonstrated that the classification accuracy of the MCS group was comparable to that of healthy individuals, whereas the VS group exhibited significantly lower accuracy than the MCS group. These findings align with previous studies employing combined EEG and fMRI techniques to assess the depth of consciousness [40, 41].

### 4.1 EEG feature during MI task

EEG_ERD reflects changes in neural synchronization during specific event‐ or task‐related processes. Brain response patterns to external stimuli and the degree of neural synchronization vary significantly across different states of consciousness [42]. For example, HC exhibits greater sensitivity to stimuli, leading to a pronounced decrease in EEG_ERD, which indicates effective functional reorganization and information processing within the neuronal network. In contrast, patients in MCS demonstrate relatively weaker brain function, resulting in a reduced response to stimuli. This study employed ERD, an energy‐based index, to classify subjects into three groups based on their performance in left‐ and right‐handed MI tasks. The results showed that the left‐handed MI task effectively distinguished between the VS and MCS groups. Whereas the right‐handed task failed to differentiate them. Since all subjects in this study were right‐handed, the findings suggest a significant energy difference in the nondominant hand of VS subjects compared to MCS subjects during the left‐handed MI task. This observation aligns with previous studies [43] and holds potential significance for DoCs. These findings further support EEG_ERD as a promising biomarker for distinguishing different states of consciousness.

Furthermore, changes in EEG_ERD are closely associated with functional connectivity between different brain regions. During conscious activity, various brain regions collaborate to facilitate information transmission and integration through synchronized neuronal discharge. EEG_ERD captures the dynamic variations in functional connectivity between these regions, and distinct consciousness states exhibit different connectivity patterns, leading to corresponding changes in EEG_ERD. This relationship aids in uncovering the neural mechanisms underlying consciousness differentiation [44]. From a neurophysiological perspective, EEG_ERD reflects the balance between excitatory and inhibitory neuronal activity. Alterations in consciousness states influence physiological processes such as neurotransmitter release and ion channel regulation, subsequently affecting neuronal activity and synchronization. These changes are ultimately manifested in EEG_ERD variations, providing a robust theoretical foundation for its use as a biomarker of consciousness states. Investigating the functional connectivity of brain networks in MI tasks will be the focus of our future research to further elucidate global brain changes.

Consequently, EEG_ERD alterations are less pronounced in MCS patients compared to HC, while patients in a VS exhibit even weaker EEG_ERD responses due to further impaired brain function. This sensitivity to varying levels of consciousness underscores EEG_ERD as a key indicator for distinguishing consciousness states. In clinical practice, accurately assessing a patient's level of consciousness is essential for developing treatment strategies and evaluating prognosis.

### 4.2 fNIRS feature during MI‐task

Numerous studies have demonstrated that increased activation of specific brain regions during task execution leads to a rise in the relative concentration of HbO [45, 46]. This phenomenon is hypothesized to result from the heightened oxygen demand associated with enhanced functional brain activity. While oxygen supply to task‐related brain regions increases accordingly [47], the simultaneous elevation in functional brain activity also intensifies oxygen consumption. Therefore, fluctuations in HbO concentration during task performance are determined by the interplay between oxygen supply and consumption. For instance, in motor imagery tasks, contralateral brain regions exhibit greater activation than the ipsilateral side during left‐hand imagery, leading to higher oxygen consumption in those regions [48]. The increased oxygen demand in task‐relevant brain regions enhances the blood's oxygenation capacity, causing a substantial rise in the relative HbO concentration. This elevation exhibits a saturation threshold: upon reaching maximal HbO concentration, its impact on HbO concentration in both ipsilateral and contralateral movement‐related regions becomes comparable. At this stage, oxygen consumption emerges as the primary determinant of HbO concentration changes. Furthermore, during task execution, total hemoglobin concentration in task‐related brain regions reflects their activation level, with higher concentrations indicating greater activation.

### 4.3 Multimodal BCI feature

Through feature importance analysis, we identified five key discriminative factors for consciousness state differentiation: fNIRS_ACC, EEG_ACC, fNIRS_slope, fNIRS_centroid, and EEG_ERD. For fNIRS, slope and centroid are indicators representing the reaction rate, and their values can indicate the level of consciousness to some extent. The consciousness level of HC is higher than that of the MCS, which is higher than that of the VS. The three groups showed different reaction rates in executing commands in response to sound stimuli. There were significant differences in differentiating different consciousness levels, while there were no significant differences in the mean values and integral values.

A single diagnostic indicator often has limitations. However, ACC, as a comprehensive classification indicator, integrates various physiological and behavioral characteristics related to consciousness. It can systematically classify information from various aspects, such as patients' performance in different tests, neuroimaging data, and clinical observation results, thus greatly improving the accuracy of diagnosing the consciousness states of the three types of patients [49]. For example, by combining EEG and fNIRS data, ACC can analyze and classify patients' brain activities from multiple dimensions, avoiding diagnostic biases caused by misjudgments of a single indicator [50]. Therefore, the research of evaluating the consciousness level from multiple perspectives by combining the EEG–fNIRS multimodal BCI paradigm, provides more reliable diagnostic references for clinicians and is expected to become a new clinical diagnostic method.

### 4.4 Limitations of the Sample

As patients with DoCs represent a unique population, recruiting sufficient participants remains a challenge. Future studies should aim to expand the study cohort to include individuals with varying degrees of brain injury and functional impairment. This broader population will facilitate a more comprehensive investigation of neurovascular changes and functional brain connectivity associated with multimodal neuroimaging. Such research will provide stronger evidence to support clinical applications and improve patient management strategies.

## 5 Conclusion

This study confirmed that the integration of multimodal EEG‐fNIRS and MI‐BCI models significantly improved the accuracy of consciousness assessment in patients with DoC. The EEG‐fNIRS multimodal framework yielded a higher diagnostic accuracy (97.47% in healthy controls, 93.28% in MCS, and 76.19% in VS) and a statistically significant improvement in classification performance (*p* < 0.05 for all patient groups) compared to the single‐modal approach. As a biomarker, EEG_ERD can be effectively distinguished between the MCS group and the VS group, which may provide an effective reference for the realization of preclinical diagnosis of consciousness disorders in the future, and solve the key limitation of misdiagnosis of subjective behavior in current clinical practice. EEG‐fNIRS biomarkers enable robust differentiation between states of consciousness, particularly through ACC parameters and task‐specific cortical activation patterns.

These findings advocate for a paradigm shift in DoC evaluation protocols; multimodal neuroimaging should be incorporated as a mandatory adjunct to CRS‐R evaluations. MI‐BCI paradigms provide an objective framework for detecting residual cognitive MI. Machine learning‐driven fusion of EEG and fNIRS features optimizes diagnostic specificity. Future research should validate these findings in larger multicenter cohorts and explore real‐time implementation for intensive care unit monitoring applications.

## Author Contributions

N.W. conceptualized the study, drafted the manuscript, participated in the design of the figures, and conducted a literature review on fNIR. X.C. and J.S. assisted in editing and revising the manuscript. Y.H., Q.H., and T.Z. conducted focused literature reviews on neuroimaging methodologies and contributed to the editing process to enhance coherence and accuracy. D.L., J.L., and T.C. provided feedback on clinical implications and contributed to data interpretation. S.Z. and Y.J. statistically analyzed the raw data of the article. J.S., W.M., Y.Y., and J.Z. supervised the study, offering substantial guidance on the research direction, critically reviewing the manuscript for intellectual content, and approving the final version for submission.

## Ethics Statement

The authors confirm that any aspect of the work covered in this manuscript that has involved patients with disorders of consciousness has been conducted with the ethical approval approved by the ethics committee of Beijing Tiantan Hospital (KY2024‐043‐03) and the Chinese Clinical Trial Registry. This study follows the guidelines of the Declaration of Helsinki for humans.

## Conflicts of Interest

The authors declare no conflicts of interest.

## Supporting information


**Table S1:** Brodmann Area of fNIRS.

## Data Availability

Data are available upon reasonable request. The original data are not yet openly available, as it is being used in ongoing projects. We welcome enquiries about sharing this as part of a collaboration, please contact the corresponding authors.
